# Getting to the heart of transformation

**DOI:** 10.1007/s11625-021-01016-8

**Published:** 2021-08-14

**Authors:** Coleen Vogel, Karen O’Brien

**Affiliations:** 1 Global Change Institute, University of the Witwatersrand, Johannesburg, P. Bag 3, Wits University, 2050 South Africa; 2 Department of Sociology and Human Geography, University of Oslo, Blindern, P.O. Box 1097, 0318 Oslo, Norway

**Keywords:** Transformation, Sustainability, Transdisciplinarity, Transgressive, Transcendence

## Abstract

Climate change, biodiversity loss, the COVID-19 pandemic, and growing inequity and poverty are some of the key global challenges facing us today. These multiple and interacting crises have elicited growing appeals to the need for transformation. Yet while the scholarly literature on transformations is expanding rapidly, the concept risks becoming an empty buzzword or an alibi for superficial interventions and business-as-usual responses within research, policy and practice communities. In this perspective, we look more closely at what is needed to generate the deep and enduring changes that are called for to address multiple, interacting challenges. We do this by focusing on the prefix ‘trans-’, which signifies moving “across, over, or beyond” the current state of affairs, and we consider how the potential for equitable and sustainable transformations lies in our capacity to transcend entrenched boundaries and limits. Focusing on transdisciplinary, transgressive, and transcendent approaches, we reflect on how individuals, groups, and organizations can plant seeds and help to nurture the potential for radical transformative change at all scales.

“A good question is never answered. It is not a bolt to be tightened into place but a seed to be planted and to bear more seed toward the hope of greening the landscape of idea.”

— John Ciardi

## Introduction

The world is in a precarious situation today, mired in ‘wicked problems’ that are draining people of hope about a brighter future. Buffeted by the COVID-19 pandemic, growing poverty and inequality, violations of human rights, and a weakening of democracy, global environmental change issues such as climate change, biodiversity loss, and marine pollution are increasingly overshadowed by day-to-day concerns about health insecurity, economic insecurity, and food insecurity. It is clear that piecemeal and “business as usual” responses are insufficient and that the challenges are cross-scalar and require attention to a range of aspects, including social, cultural, economic, political, institutional, demographic, psychological, behavioral, and technical dimensions (IPBES 2019).

The signals regarding the growing scope, scale, and urgency of the problem are clear and require just, sustainable, and (re)generative change (Pereira et al. 2020; Waddock et al. 2020). This requires new, collaborative approaches to knowledge systems and narratives of change, yet it also calls for radical and emancipatory political change (Fazey et al. 2020; Veland et al. 2018; Wright 2013). An open question is whether humans can respond to these interconnected issues effectively, before tipping points are reached, planetary boundaries are further exceeded, collective traumas increase, and future opportunities are foreclosed (Steffen et al. 2015; 2018; Brulle and Norgaard 2019). Can we transform our societies rapidly and generate an equitable, inclusive, and sustainable world? The answer to this question is anything but clear. Although the scholarly literature on transformations is expanding rapidly, the concept risks becoming an empty buzzword or an alibi for superficial or business as usual responses in research, policy, and practice (Blythe et al. 2018). There is growing attention to the need to delve deeper and consider how to nurture transformations by “taking diverse knowledges seriously,” “taking politics seriously,” and “taking plural pathways seriously” (Scoones et al. 2020, 70).

In this commentary, we look more closely at what is needed to generate the deep and enduring changes that are called for to address multiple, interacting challenges. We do this by focusing on the prefix ‘trans-’, which signifies moving “across, over, or beyond” the current state of affairs. In particular, we consider how the potential for equitable and sustainable transformations lies in our capacity to go beyond established and entrenched boundaries and limits. Drawing attention to transdisciplinary, transgressive, and transcendent approaches, we reflect on the ways that individuals, groups, and organizations can plant seeds and help to nurture the potential for ‘trans-’ “formations” at all scales.

## Transformation as trans-formation

Transformation has been defined and described in many ways, and the academic literature is expanding rapidly (Feola 2015; Salomaa and Juhola 2020). The science justifying the need for rapid, radical change has also been growing, but what is now required is more than knowledge and facts. There is a recognized need for social transformations that simultaneously involve and engage with the practical, political, and personal spheres (O’Brien 2018). This calls for creative, imaginative, and experiential ways of thinking, communicating, and generating change, and draws attention to the role of the arts and storytelling (Galafassi et al. 2018; Veland et al. 2018; Tosca 2019). It requires opening our individual and collective’heart’ and expanding our circles of care, at the same time engaging in alternative approaches to politics (Adnan 2021).

The potential of individuals and organizations to consciously transform themselves and their societies is described by Ziervogel et al. (2016, 2) as a “capacity to imagine, enact, and sustain a transformed world and a way of life that is in balance with the carrying capacity of our earth, and where all life flourishes.” This deeper transformation represents “a process of becoming,” which is considered by Berzonsky and Moser (2017, 17) to be at the heart of transformation. Yet how do we unleash these capacities for transformative change? Ziervogel et al. (2016) identify three key aspects of transformative capacity: an awareness of and reconnection to visible systems that support wellbeing; a well-developed sense of agency; and strong social cohesion. Research shows that these capacities and characteristics can be nurtured and developed, including through mindfulness (Wamsler et al. 2018; Schlitz et al. 2010). However, although psycho-cultural components of transformation are important, there is a risk of getting stuck in mental approaches and the psychology of “how we think,” which may divert attention from ways of activating political and collective agency for systems change.

**Fig. 1:**
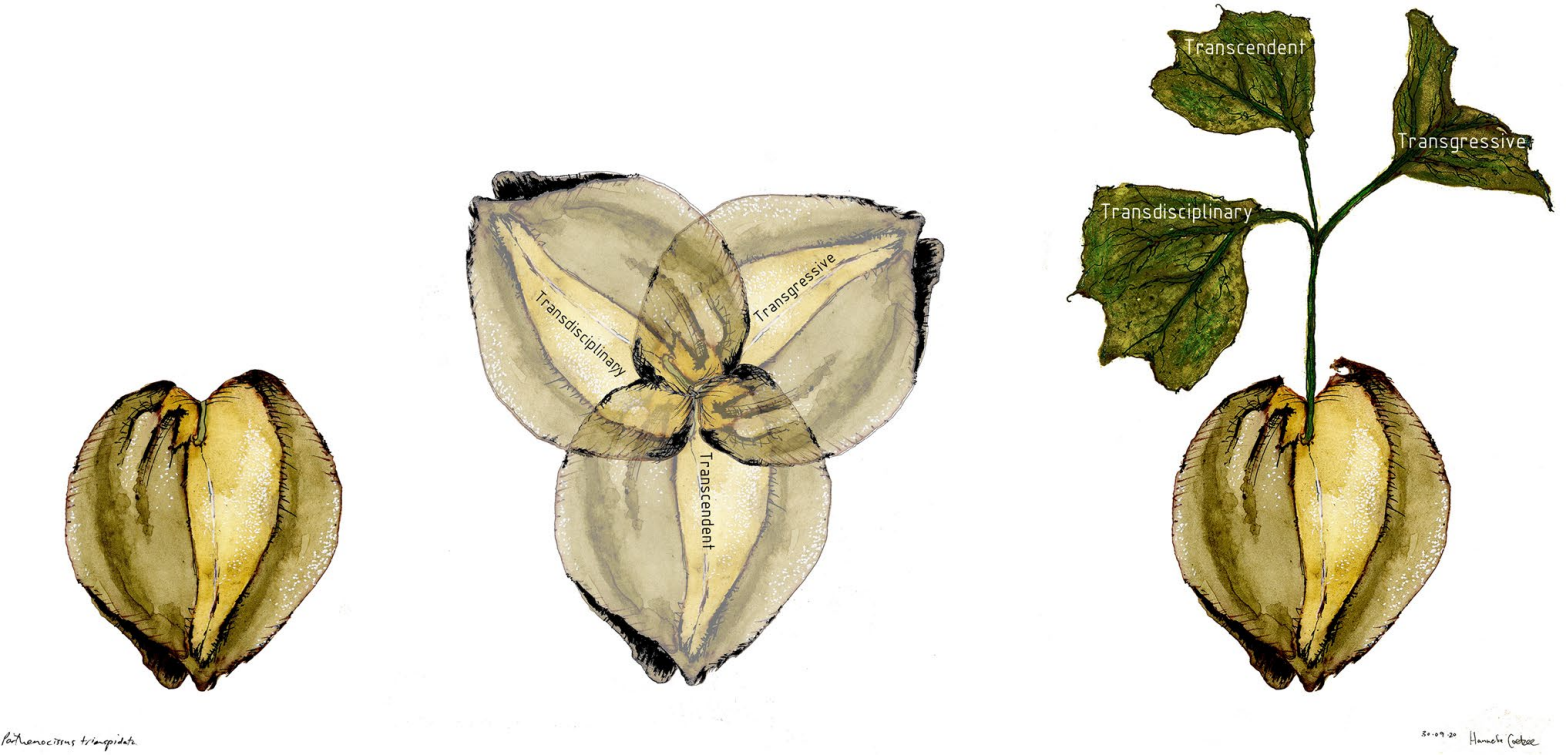
The heart of transformation: seeding and nurturing change

Below, we discuss three entry points for “seeding” transformations that may take us above, across and beyond current ways of being and doing, namely transdisciplinary, transgressive, and transcendent approaches (Fig. 1). The seed is an appropriate metaphor, as it symbolizes potential and hope. The concept of seeds has also been used to describe emerging sustainability initiatives that are not currently dominant or prominent in the world (Bennett et al. 2016). Seeds can be sown and seedlings can be nurtured to realize the potential for transformations that are practical, political, and personal (O’Brien 2018). We consider transdisciplinary, transgressive, and transcendent approaches to be mutually interactive and supportive, and to encompass principles that can seed equitable transformations to sustainability.

### Transdisciplinary approaches

There is no single knowledge system, method, or approach that can spur the diversity of actions needed to transform across scales (Escobar 2020; Scoones et al. 2020). A transdisciplinary approach, however, can assist in supporting transformations by taking diverse types of knowledge seriously. Transdisciplinary approaches can help us move across intellectual and disciplinary silos and inspire new thinking that helps groups to both uncover and discover novel solutions to complex challenges. In describing transdisciplinarity as an educative process, McGregor’s (2015b, 9) notes that it is “concerned with creating new, integrative knowledge to address the complex problems of *the world*.” Rather than seeking “manuals,” “roadmaps,” or “blueprints,” transdisciplinary approaches and practices integrate perspectives and focus on processes and relationships that increase engagement and traction with policy makers, practitioners and other actors, including citizens.

Becoming more acutely aware of the importance of Nicolescu’s (2010) concept of “the included middle” can help to get to the heart of transformation and shift approaches from a ‘fixing’ and technical frame towards a more explorative mode of approaching challenges. “The included middle” can be interpreted as a fecund middle ground where knowledge is open, emergence is held, inclusive logic is respected, and tolerance in contradictions can be explored (McGregor’s 2015a). Transdisciplinary approaches, if carefully seeded and nurtured, can enable people from diverse contexts and backgrounds to enter McGregor's (2015b) “fecund middle ground,” which includes culture, art, religion, and spirituality. It is a zone of non-resistance that is “ripe with potentialities” and can allow new mindsets and ways of seeing the world. Such fecund spaces represent more than opportunities for dialogues where “stakeholders” are gathered for inputs; they are carefully crafted spaces where people can discuss, debate, and co-create complex futures (Charli-Joseph et al. 2018; Pereira et al. 2020).

Within these spaces, what is currently not visible, what is often impossible to talk about, and what is absent and contradictory in everday discussions can emerge (Andreotti et al. 2015). At the same time, we should be wary of what Lynch and Veland (2018, 137) refer to as “the false haven of consensus, the mirage of the win–win” that dominates much of the sustainability discourse. In other words, transdisciplinary approaches are not merely about including more voices around the table, or including non-academic stakeholders in the process of knowledge production (Rigolot 2020), but about probing and relating to different perspectives, which can open up new imaginaries and possibilities of change.

Transdisciplinary approaches engage with deeper perspectives on social change, including wisdom traditions and indigenous knowledge (Waddock et al. 2015; 2020 Johnson and Murton 2007; Grincheva 2013; Gram-Hanssen et al. 2021). Indeed, Grincheva (2013, 15) emphasizes that it is “imperative to understand, acknowledge, recognize, and appreciate epistemic cultures originating from various historical, social and cultural backgrounds, because these various epistemologies can significantly enrich the nature of human research enquiry and enhance our harmonic world perception.” Explorations of local, tacit, and indigenous knowledge systems can be seen as a deeply humble approach to sustainability, and it invites us to co-explore how we can more coherently ‘see’ our role in the complex worlds in which we are living.

Transdisciplinarity is thus an approach, a process, a practice, and a capacity that draws attention to the quality of relationships. It involves being respectful of various ways of knowing and perceiving what is real. It can be considered a *way of being.* As Rigolot (2020, 4) points out, “[w]hen transdisciplinarity is taken as a way of being, the need for knowledge and know-how for integration and implementation extends far beyond the scope of research projects and appears constantly and ubiquitously in real life.” Such an approach grounds transformations in everyday experiences, and it often raises tensions around power differentials and the contestation of power (Rigolot 2020).

### Transgressive approaches

As *a way of being*, transdisiciplinarity can in some cases call for transgressive approaches to transformations, particularly if the goal is to enhance equity, social justice, and well-being for society at large (Rigolot 2020; Bennett et al. 2019). Transgressive approaches recognize the existence of power asymmetries, where the interests of some dominate at the expense of the well-being of others. *Transgressive* refers to actions that involve a violation of moral or social boundaries, and they include a disruptive element that recognizes the many ways that most contemporary systems (e.g., social, economic, agricultural, and energy systems) are misaligned with equitable and sustainable development pathways. Transgressive approaches often involve overstepping, going against the grain, and moving beyond the current constraints to socially just actions, institutions, and change (Ziervogel et al. 2016). This takes the politics of transformation seriously, recognizing the need for thorough rather than superficial change.

Transgressive approaches to transformations challenge what is presented as ‘a given’ and surfaces the normative framings behind those givens, pushing for a re-evaluation and re-imagination of the status quo. They can help to challenge the systems and structures that create risk and vulnerability in the first place, as well as the mindsets and interests that perpetuate them (Kaika 2017). In *Pedagogy of the Oppressed*, Paolo Freire (1970) emphasized the importance of challenging the taken-for-granted nature of reality in working for enduring social change. He warned that “[t]he more completely the majority adapt to the purposes which the dominant minority prescribe for them (thereby depriving them of the right to their own purposes), the more easily the minority can continue to prescribe” (Freire 1970, 70). Teaching critical thinking is a core aspect of social change, and actively preparing for transformation thus also includes ‘transgressive’ changes to curriculum at primary through tertiary levels (Lotz-Sisitka et al. 2016).

Transgressive approaches to social change can be considered a capacity to transcend the socialized mind, and to disrupt the ‘isms’ that keep society divided, whether racism, classicism, elitism, sexism, extractivism, or any other exclusionary ideology (Kegan and Lahey 2009; Kaika 2017; Sharma 2017). The significance of time, context, and situated knowledge and right relations (e.g., decolonialisation, gender rights) remain critical when trying to transgress current ways of doing things (Andreotti et al. 2015; Gram-Hanssen et al. 2021). Importantly, transgressive approaches can also involve caring and cautious acts, so that well being and social change are imbued with learning and humility (Lotz-Sisitka et al. 2016).

### Transcendent approaches

Both transdisciplinary and transgressive approaches often call for a capacity to *go beyond* the range or limits of existing capacities, including the persistent difficulties of trying to decide or establish whose view of the world is ‘right.’ Such capacities involve the discovery of new logics, a willingness to view things from new perspectives, and an openness to shifts in meaning-making. This brings us to perhaps one of the most important aspects of transformations – namely, *transcendence*. To transcend something involves going beyond the usual conceptual understanding or human experience. It does not mean ignoring the prevailing context, conditions, contradictions and grievances, nor does it mean to accommodate the status quo. However, it does involves developing a ‘perspective on perspectives,’ which includes, for example, being able to look *at* beliefs and paradigms, rather than *through* them (O’Brien 2021).

Collectively, this means designing and engaging with systems changes at all scales, without familiar blue prints or roadmaps, but by allowing many solutions to grow and thrive. This may involve, for example, not only implementing Climate Action Plans or “Doughnut Economics” at the local, city, and national scales, but also inviting in new ideas, new ways of seeing the world, and new practices at smaller scales, including ‘circles’ and agoras, citizens and translocal assemblies, and other spaces of action within diverse and dynamic contexts (Raworth 2017; Adnan 2021). Being able to move forward, even when the pathway is not clear, requires imagining alternatives, including an alternative politics and alternative ways of planning and managing for the future (Milkoreit 2017).

Transcendent approaches can support the “open, plural and democratic politics” that are widely considered necessary for transformative change (Scoones et al. 2020, 69). As Wright (2013, 22) argues, “A vision of emancipatory alternatives anchored in the multidimensional and multiscalar problem of deepening democracy can encompass this wide range of strategies and projects of transformation.” Yet transcendence towards an emancipatory politics involves more than an undoing; it also enables the emergence of something new and unprecedented (Wilber 2000). From the current situation, the very idea of transformations to an equitable and sustainable world represents what Wilber (2000, 189) describes (within a different context) as “a new, emergent, and unprecedented endeavor,” where even “the possibility itself is an *emergent*—it *never* was, but is now coming to be—and so we do not need to wildly reinterpret the past to find hope for the future.” While learning from the past and from a pluriverse of cultures and contexts is important (Escobar 2018), the transformations that are called for right now are also likely to be generative and emergent.

Yet the possibility and potential for transcendent approaches to transformations cannot be simply left “to emerge” within complex systems. Rather, it involves activating human agency and capacities to generate such changes. This includes not only individual agency, but also collective agency and political agency (Otto et al. 2020; Maiguashca and Marchetti 2013). To activate agency and realize transformative capacities will no doubt move some people out of their comfort zones, as the liminal, fecund space of transformation is unfamiliar to many. Yet as Berzonsky and Moser (2017, 17) note, “Inherent in any becoming, however, is an ending which begins the severance, the separation from the previous world. The transformative process could not begin if the subject were not agreeing to destabilize itself in service to change.”

Developing the capacity to transcend current ways of being and doing will require different energies and skills, and the challenges of doing such work are well known (Ziervogel et al. 2016; Göpel 2016). In addition, there is a growing recognition of the emotional toll experienced by change agents (Head 2016; Macy and Brown 2014). Indeed, it can be disheartening to experience outcomes associated with fragmented societies and the disconnections between humans from nature. Supporting communities of practice and creating spaces where help and support can be gathered and nurtured is thus also essential. Certainly, the seeds for rapid and deliberative change are already being sown (Bennett et al. 2016), and the capacity to transform is indeed being nurtured. As individuals and groups begin the transformative journey, a number of tools and practices are becoming available to support these *trans*formations, whether this refers to bridging across, stepping over, or moving beyond (e.g., Pereira et al., 2019; Galafassi et al. 2018). However, to accelerate these transformations at scale involves engaging individuals, groups, and organizations of all types with transformative politics and practices.

## Conclusion: The heart of transformation

The question of how to ‘unfold’ individual and collective capacities for transformations to an equitable and thriving world demands that we get to the heart of the issue. In this brief commentary, we have considered how transdisciplinarity, transgressive and transcendent approaches can contribute to the unfolding of these capacities and spaces, which in turn can influence research, policy, and practice. Transformationis is simultaneously practical, political, and personal. The ‘heart of transformation,’ we argue, involves going beyond our current ways of being and doing and embracing the unfolding of humanity’s collective capacity and potential to collectively shift systems and cultures, while also ensuring that transformations are equitable, inclusive, and not the least, sustainable. Individually, this includes a recognition that everyone holds the capacity to generate transformations to a thriving world (Sharma 2017). It also involves the recognition that real transformations will be resisted and subverted (Brand 2016; Williamson 2012), and that a combination of transdisciplinary, transgressive, transcendent, and other ‘trans-’ strategies are needed to overcome such resistance and generate radical, equitable, and enduring change.

Transformation is, at its heart, a deeply holistic, reflective, and relational process. As Terry Patten (2018, 150) writes, “wholeness is the most primary, root quality of existence, and the heart is where wholeness is intuited—and love is its expression.” Patten also points out that wholeness is more deeply real than the fragmentation, separation, and division that cognitive minds perceive, and that “wholeness is transmuted at the heart into our wisest feeling-impulses – like care, appreciation, well-being, affection, strength, and courage” (Patten 2018, 73). Transformations thus involve activating “the intelligence of the heart, not only in the individual, but also in the collective” (Scharmer 2016). Sharma (2017) describes this space of wholeness and the innate universal values associated with it—such as equity, dignity, and compassion—as a powerful source for radical transformations. An awareness and mindfulness of such values can help to unfold and nurture the potential for change that already exists in everyone, i.e., the seeds for a thriving world.

Although there are no one-size-fits-all solutions to today’s complex problems, the seeds for transformation can be continuously sown, nurtured and developed. As we are reminded in the epigraph by poet John Ciardi, the question of *how* to transform is not meant to be answered. Instead, we can sow and nurture the seeds for “greening the landscape of idea,” including the idea that transformations are, in fact, possible. Yet the process and practice of transformation involves our individual and collective willingness to go beyond current ideas of social change.
